# The first generation ABSORB BVS scaffold; to be or not to be?

**DOI:** 10.1007/s12471-017-1007-y

**Published:** 2017-06-22

**Authors:** J. P. S. Henriques, J. Elias

**Affiliations:** Department of Cardiology, Academic Medical Center – University of Amsterdam, Amsterdam, The Netherlands

In the routine treatment of coronary artery disease, balloon angioplasty has been replaced by stents. The use of bare-metal stents resulted in lower rates of restenosis and repeat revascularisations. Drug-eluting stents (DES), designed with thinner struts and anti-proliferative drugs, have further enhanced the efficacy and safety of percutaneous coronary intervention. Nevertheless, the new generation of metallic DES are also limited by risks of neoatherosclerosis with late and very late stent thrombosis and a rigid metallic cage impairing vasomotion [1].

Bioabsorbable ‘stents’ were developed to restore coronary flow and temporary support the vessel with the advantage of dissolving completely over time, possibly overcoming abovementioned short-comings of metallic DES. The ABSORB (Abbot Vascular, Santa Clara, California, USA) bioabsorbable vascular scaffold (BVS), consisting of a poly-L-lactide backbone coated with a mixture of poly-D,L-lactide and an everolimus-eluting drug, was the first bioresorbable device to receive CE (Conformité Européene) approval in 2010 and approval by the Food and Drug Administration in the United States in 2016. Studies performed in relative simple coronary lesions have shown reasonable short-term to mid-term results. Conversely, world-wide registries from clinical practice raised concerns regarding increased rates of scaffold thrombosis.

Recently, longer-term follow-up randomised clinical data have become available. The ABSORB II trial is the first randomised trial reporting 3 years’ follow-up. This trial did not meet the co-primary endpoints: BVS compared with Xience stent did not show superiority on vasomotion and failed the non-inferiority on angiographic late luminal loss. The device-oriented endpoint, consisting of cardiac death, target-vessel myocardial infarction and revascularisation, was higher in the BVS group. There were no differences between both arms for angina and exercise capacity. A striking ongoing risk, from 1 up to 3 years, of scaffold thrombosis was observed with a very late scaffold thrombosis rate of 2%, whereas in the Xience arm no patient experienced very late stent thrombosis [2]. Furthermore, the ABSORB III trial, presented at ACC 2017, showed a significantly higher two-year target lesion failure in the BVS compared to Xience, leading to a FDA safety alert on the performances of BVS.

The AIDA investigator-initiated trial is the first, large, randomised study comparing the BVS with Xience in a patient population reflecting routine clinical practice. Results of the trial were reported earlier based on a recommendation by the Data and Safety Monitoring Board due to safety concerns. The study reported a higher rate of scaffold thrombosis throughout two years. Still, mortality rates, all-cause and cardiac, and repeat revascularisation were not significantly different between the two groups [3].

In our meta-analysis of 7 randomised clinical trials including 5577 patients in this issue of the Netherlands Heart Journal, we describe a higher target lesion failure in BVS when compared to the Xience [OR 1.34; 95% CI 1.11–1.62, *p* = 0.003]. Additionally, a highly significant increased risk for definite and probable device thrombosis was observed in BVS when compared with Xience. Furthermore, our meta-analysis demonstrates with a landmark analysis an ongoing higher risk of device thrombosis throughout the follow-up, even after excluding the early events [4].

Currently, the exact mechanism underlying these higher risks of scaffold thrombosis is only partially understood. The poorer acute and subacute results have been attributed to suboptimal implantation techniques. BVS-specific implantation protocols, consisting of adequate pre-dilatation, proper sizing and post-dilatation, may address incomplete BVS expansion. A post-hoc analysis suggested that these protocols could reduce scaffold thrombosis by 70% to rates comparable with metallic DES [5]. Possible explanations for the reported late and very late scaffold thrombosis might be heterogeneous endothelialisation of scaffold struts and incomplete integration into the vessel wall. Uncovered scaffold struts can cause late strut discontinuity and protrusion of struts into the lumen triggering thrombus formation. Also, a longer than anticipated resorption process and resorption-related scaffold discontinuity in response to vascular inflammation may lead to these late events [6]. Although it is unlikely that implantation techniques are solely associated with late onset events, they may theoretically still be related, as not all resorption-related issues are understood.

In view of the ongoing risk for scaffold thrombosis, beyond 12 months, the AIDA investigators proposed and implemented prolongation, and reinstallation, of dual anti-platelet therapy (DAPT) in all BVS patients, including the patients outside AIDA, unless contra-indicated. It is unknown if prolonged DAPT can prevent very late BVS-related thrombotic events. However, prolonged DAPT reduced thrombotic events and was safe in the DAPT and PEGASUS trials. The ongoing COMPARE ABSORB, aiming to enrol 2100 high-risk patients, has also allowed DAPT exceeding 12 months.

After publication of the AIDA data, the Netherlands Society of Cardiology (NVVC) advised all cardiologists in the Netherlands to prolong DAPT up to three years of follow-up. On the 31^st^ of March 2017, Abbott Vascular discontinued normal commercial sales of the Absorb BVS and limited its usage in Europe to clinical registries and studies in order to monitor implant technique. In light of current developments, Everaert and colleagues updated the 2015 Dutch position statement on the appropriateness of BVS in PCI in this issue of the Netherlands Heart Journal. In their new 2017 recommendations, the use of current BVS is downgraded and limited to dedicated registries using the updated implantation protocol. They also advise to prolong DAPT in patients with a high risk of ischaemic events [7].

Results from recent trials have divided the interventional field on the use of BVS into ‘believers’ and ‘sceptics’. The sceptics do not welcome BVS usage in daily practice because of more difficult placement and inferior efficacy and safety. Believers hold on to a future benefit of the scaffold. They believe that inferior outcomes are driven by lack of experience and suboptimal implantation techniques. Nonetheless, scaffold thrombosis leads to worse patient outcome. Putting patients at an increased risk should only be considered if the advantages are superior to the best-in-class (Xience DES) treatment. Currently, no clear evidence supports the hypothetical future benefits of the BVS, but this could still be due to a longer than anticipated resorption process.

The current data put the overall picture of BVS for routine clinical practice in perspective. We cannot simply state that the start-up phase with the BVS resembles the introduction of the first-generation DES, which also had a higher incidence of acute and late stent thrombosis. We should not forget that the higher incidence of stent thrombosis in the first-generation DES was offset by a strong reduction of in-stent restenosis with less repeat target lesion and vessel revascularisations. In short, there was at least some clinical benefit. In the case of BVS, such benefit has not been proven yet.

There are concerns of early and late, and very late, thrombotic events in patients treated with the Absorb BVS and these events are associated with worse outcome. However, these events are rare and do not translate into a higher mortality when compared with Xience. In theory, bioresorbable scaffolds hold a great promise. The overall available clinical data on the current device do mandate caution, but – of course with new insights and future technical advances – the BVS could still be the next revolution in coronary artery treatment. For routine PCI in clinical practice, however, the first-generation ABSORB scaffold seems ‘not to be’.
